# Minimally invasive technique cuts costs in veterinary surgery

**DOI:** 10.4103/0972-9941.16535

**Published:** 2005-06

**Authors:** V. M. Chariar, D. N. Shirodkar, Y. B. Khare

**Affiliations:** Honorary Laparoscopic Surgeon, Stray Dog Sterilization Center, Veterinary Department, Thane Municipal Corporation, Thane; 1 Chief Veterinarian, Veterinary Department, Thane Municipal Corporation, Thane; 2 Veterinary Surgeon, Paws – Doc Speciality Small Animal Medicine & Surgery, Mumbai, India

Dear Editor,

Urban stray dog population control has been a pressing and ever-present challenge in India. Euthanasia or conventional sterilization techniques have by and large failed to stem this problem. Zoonotic disease and aggressive behavior displayed by packs of feral stray dogs remain areas of concern.

A stable, sterilized and, where possible, immunized stray population is the need of the hours, to ensure peaceful coexistence between the man and the animal. The Thane municipal corporation's (TMC) stray dog sterilization center was set up in January 2004 with this aim in mind. The center uses minimal access surgery for neutering female dogs and conventional open surgery for male dogs.

In a developing country like India, availability of limited resources inevitably fuel doubts about the need for and appropriateness of a technology-intensive solution to such a problem. Investment in laparoscopic instrumentation, video cameras, and suction-irrigation and light systems may be easily dismissed as an unaffordable luxury. The center represents a compelling case for the need for using minimal access technology in the neutering program for stray dogs.

**1. Rapid turnover of operated bitches:** the center has 36 kennels and has operated 3519 dogs in its first 333 days, amounting to 3.41 kennel days per dog. Stray bitches are released on the third day following surgery. This is in contrast to the average 12.17 kennel days per dog undergoing conventional open neutering surgery (a typical 100 kennel center operates a maximum of 3000 dogs in a calendar year). In the larger perspective of a stray dog sterilization program, every kennel day saved could mean the difference between success and failure, given the exponential rate of population growth. The certainty of wound healing from the mechanical stability of 5-mm puncture wound closures is a key factor in the success of this program.

**2. Benefit to patients:** the well-documented patient benefits of reduced surgical trauma, reduced operative time, reduced morbidity, and reduced pain were central to the rapid recovery, return to fitness and confidence for release of neutered stray bitches.

**3. The economic reality:** medicine and distributed cost of equipment amounted to Rs. 305 per surgery and compares favourably with that spent for conventional surgery. At other centers funds from nongovernment organizations augment a subsidy of Rs. 340 provided by the animal welfare board of India for each such surgery.

Thus, in the longer term the one time cost involved in providing for laparoscopic infrastructure is far more economical as compared to the consistently higher running costs of conventional surgery.

We have thus established the indispensability of minimal access technology. Given the shoestring budgets allocated to the stray dog-neutering program in a developing country like ours, it would be heartening to see this technique proliferate and provide value in each urban zone in the country.

